# P-411. Activity of Omadacycline Against 35,000 Bacterial Clinical Isolates and Resistant Organism Subsets from Patients in the United States (2019–2023)

**DOI:** 10.1093/ofid/ofae631.612

**Published:** 2025-01-29

**Authors:** Michael D Huband, Kelley A Fedler, Kelly Wright, Rodrigo E Mendes, Mariana Castanheira

**Affiliations:** Element, North Liberty, IA; Element, North Liberty, IA; Paratek Pharmaceuticals, Inc., king of Prussia, Pennsylvania; Element, Iowa City (JMI Laboratories), North Liberty, IA; JMI Laboratories, North Liberty, Iowa

## Abstract

**Background:**

Omadacycline (OMC) is a tetracycline class (aminomethylcycline) antibacterial with potent activity against bacterial isolates expressing common mechanisms of resistance to tetracyclines, penicillins, fluoroquinolones, macrolides, β-lactams, and vancomycin. This study determined the *in vitro* activity and susceptibility of OMC against 35,000 bacterial isolates collected during the 2019-2023 OMC post-marketing surveillance program.

**Figure:**
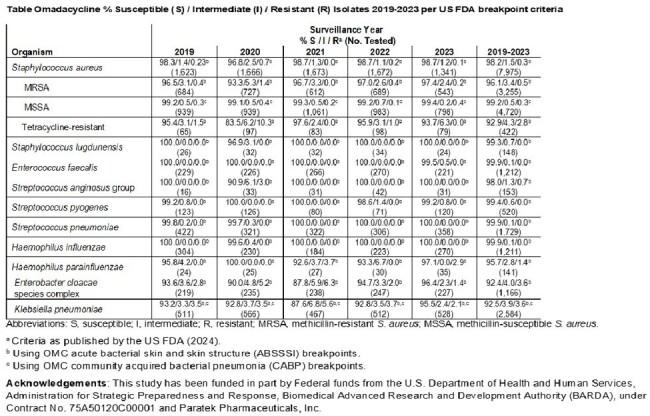

**Methods:**

Bacterial isolates were recovered from patients (multiple infection types) in medical centers in the USA from 2019–2023. Species identification was confirmed by MALDI-TOF MS. MIC testing was conducted according to CLSI M07 (2023) and M100 (2024) guidelines. OMC MIC results were interpreted using FDA breakpoint criteria.

**Results:**

Gram-positive isolates demonstrated at least 98% susceptibility to OMC. Species and phenotypes of interest including methicillin-resistant *Staphylococcus aureus* (MRSA; *n*=3,255), *S. lugdunensis* (*n*=148), vancomycin resistant *E. faecalis* (*n*=30), *S. anginosus* group (*n*=153), erythromycin-resistant *S. pyogenes* (*n*=133) and penicillin-resistant *S. pneumoniae* (*n*=193; oral breakpoint) demonstrated high susceptibility to omadacycline with 96.1%, 99.3%, 100%, 98.0%, 98.5%, and 100% susceptible, respectively. Overall Gram-negative susceptibility to OMC ranged from 92.4% to 99.9%. Gram-negative phenotypes of interest including beta lactamase positive *H. influenzae*/*H. parainfluenzae* (n=367), ceftazidime non-susceptible *E. cloacae* (n=355), and carbapenem resistant *K. pneumoniae* (n=50) demonstrated susceptibility to omadacycline of 100%, 85.8%, and 86.0% respectively. For the subset of strains that were tetracycline-resistant, *S. aureus* (*n*=422), *E. faecalis* (*n*=842), *S. anginosus* group (*n*=59), *S. pyogenes* (*n*=162), and *S. pneumoniae* (*n*=343), omadacycline susceptibility was 92.9%, 100%, 94.9%, 98.1%, 99.7% respectively.

**Conclusion:**

OMC demonstrated potent and consistent *in vitro* activity with susceptibilities of >90.0% against medically important FDA-indicated pathogens (including resistant phenotypes) during the 5-year post-marketing surveillance period (2019-2023) from medical centers in the United States.

**Disclosures:**

**Michael D. Huband, BS**, Paratek Pharmaceuticals: Advisor/Consultant|Paratek Pharmaceuticals: Grant/Research Support **Kelley A. Fedler, BS**, Paratek Pharmaceuticals: Grant/Research Support **Kelly Wright, PharmD, BCPS**, Paratek Pharmaceuticals: Employee **Rodrigo E. Mendes, PhD**, JMI: RM is an employee of JMI. JMI was contracted by and received financial support from GSK to conduct gepotidac|Paratek Pharmaceuticals: Advisor/Consultant|Paratek Pharmaceuticals: Grant/Research Support

